# Influence of weekday of admission and level of distress on length of hospital stay in patients with low back pain: a retrospective cohort study

**DOI:** 10.1186/s12891-021-04529-6

**Published:** 2021-08-05

**Authors:** Emanuel Brunner, André Meichtry, Davy Vancampfort, Reinhard Imoberdorf, David Gisi, Wim Dankaerts, Anita Graf, Stefanie Wipf Rebsamen, Daniela Suter, Lukas Martin Wildi, Stefan Buechi, Cornel Sieber

**Affiliations:** 1 Department of Physiotherapy and Rehabilitation, Winterthur Cantonal Hospital, Brauerstrasse 15, CH-801 Winterthur, Switzerland; 2 Department of Health, OST – Eastern Swiss University of Applied Sciences, St. Gallen, Switzerland; 3 Department of Rehabilitation Sciences, KU Leuven, Leuven, Belgium; 4 School of Health Professions, Zurich University of Applied Sciences, Winterthur, Switzerland; 5 Department of Internal Medicine, Winterthur Cantonal Hospital, Winterthur, Switzerland; 6 Department of Medicine, Nursing, Winterthur Cantonal Hospital, Winterthur, Switzerland; 7 Department of Medicine, Institute of Rheumatology, Winterthur Cantonal Hospital, Winterthur, Switzerland; 8 Clinic for Psychotherapy and Psychosomatics “Hohenegg”, Meilen, Switzerland; 9 Institute for Biomedicine of Ageing, Friedrich-Alexander-Universität Erlangen-Nürnberg, Nürnberg, Germany

**Keywords:** Pain management, Mental health, Primary care, Low back pain, Primary care hospital

## Abstract

**Background:**

Low back pain (LBP) is often a complex problem requiring interdisciplinary management to address patients’ multidimensional needs. Providing inpatient care for patients with LBP in primary care hospitals is a challenge. In this setting, interdisciplinary LBP management is often unavailable during weekends. Delays in therapeutic procedures may result in a prolonged length of hospital stay (LoS). The impact of delays on LoS might be strongest in patients reporting high levels of psychological distress. Therefore, this study investigates the influence of weekday of admission and distress on LoS of inpatients with LBP.

**Methods:**

This retrospective cohort study was conducted between 1 February 2019 and 31 January 2020. In part 1, a negative binomial model was fitted to LoS with weekday of admission as a predictor. In part 2, the same model included weekday of admission, distress level, and their interaction as covariates. Planned contrast was used in part 1 to estimate the difference in log-expected LoS between group 1 (admissions Friday/Saturday) and the reference group (admissions Sunday-Thursday). In part 2, the same contrast was used to estimate the corresponding difference in (per-unit) distress trends.

**Results:**

We identified 173 patients with LBP. The mean LoS was 7.8 days (SD = 5.59). Patients admitted on Friday (mean LoS = 10.3) and Saturday (LoS = 10.6) had longer stays, but not those admitted on Sunday (LoS = 7.1). Analysis of the weekday effect and planned contrast showed that admission on Friday or Saturday was associated with a significant increase in LoS (log ratio = 0.42, 95% CI = 0.21 to 0.63). A total of 101 patients (58%) returned questionnaires, and complete data on distress were available from 86 patients (49%). According to the negative binomial model for LoS and the planned contrast, the distress effect on LoS was significantly influenced (difference in slopes = 0.816, 95% CI = 0.03 to 1.60) by dichotomic weekdays of admission (Friday/Saturday vs. Sunday-Thursday).

**Conclusions:**

Delays in interdisciplinary LBP management over the weekend may prolong LoS. This may particularly affect patients reporting high levels of distress. Our study provides a platform to further explore whether interdisciplinary LBP management addressing patients’ multidimensional needs reduces LoS in primary care hospitals.

## Introduction

Low back pain (LBP) is a highly prevalent and costly health problem worldwide [1, 2]. To reduce the global burden, there is an urgent need for effective and cost-efficient strategies aiming to manage LBP at all levels of health care [3]. Most patients with LBP have a favourable prognosis regarding disability and pain [4–6] and can mostly be treated in outpatient settings. However, occasionally, patients with LBP are hospitalized in primary or acute care hospitals. In Switzerland, approximately 6% of all people with LBP are admitted to inpatient care in hospitals [2]. However, this relatively small group accounts for approximately 12% of the total direct costs for LBP in Switzerland, which are estimated at €2.6 billion annually [2].

Despite the relevant healthcare burden caused by hospitalization, inpatient care for patients with LBP remains largely unstudied. In Australia, it has been shown that patients with concurrent LBP admitted to a specialized hospital had a longer LoS than those without LBP [7]. Another study from Australia investigated inpatient LBP management in different general hospitals. The results indicate that patients with LBP admitted to general medicine units stayed longer in the hospital than those admitted to specialized rheumatology units [8]. Furthermore, Kyi et al. (2019) found that in women, age ≥ 60 years, the presence of comorbidities and a diagnosis of canal stenosis or a disc-related diagnosis were significantly associated with a prolonged LoS (> 4 days) [8]. To date, it is unclear to what extent psychological factors associated with LBP have an impact on LoS.

LBP is often multidimensional in nature due to complex interrelationships of physical, psychological, and social factors [9]. In outpatient settings, psychological factors were identified as significant predictors of poor treatment outcomes [10–12]. Therefore, it is likely that psychological factors complicate inpatient management in general hospitals. Patients with LBP desire clear, consistent and personalized information on their prognoses, treatment options and self-management strategies [13]. Unclear information or inconsistent treatment strategies in different healthcare professions may increase patient distress, fear and suffering. Therefore, providing patient-centred LBP management at primary care hospitals is a complex challenge.

Generally, patients admitted to acute care hospitals on weekends are at risk of delayed clinical management, resulting in an increased LoS [14]. Associations between weekdays of admission and LoS in acute care hospitals have never been explored in patients with LBP. We hypothesize that the influence of delayed clinical management on LoS might be strongest in patients with LBP and high psychological distress. They may respond more negatively to delays and potential uncertainties regarding diagnosis and treatment options than those with lower levels of distress.

Against this background, this study aims (1) to investigate LoS in terms of weekday of admission in patients with LBP admitted to medical units at a primary care hospital in Switzerland and (2) to explore whether the effect of the weekday of admission on LoS was moderated by patient-reported distress. We expected that patients with LBP having to wait more than 2 days for patient-centred LBP management (due to admissions on Friday/Saturday) would stay longer in the hospital than those receiving multidimensional LBP management within the first 2 days of hospitalization (admissions on Sunday-Thursday). Furthermore, we expected that patient-reported distress would moderate the effect of the weekday of admission (Friday/Saturday vs. Sunday-Thursday) on LoS.

## Materials and methods

### Data collection and clinical setting

We included all patients with LBP admitted to a medical unit at the Winterthur Cantonal Hospital during a 12-month period (February 1, 2019, to January 31, 2020). Winterthur Cantonal Hospital is a public acute care hospital with over 28,000 inpatient visits a year. In 2019, the medical units had 8216 inpatients [15].

We identified patients with LBP admitted to medical units on the screening list. All patients received pencil and paper questionnaires for assessing health-related personal data at the initial physiotherapy consultation. We asked patients to complete the questionnaire and sign the informed consent document concerning the use of health-related personal data for research purposes. Patients returned the completed questionnaires to their physiotherapist.

**Fig. 1:**
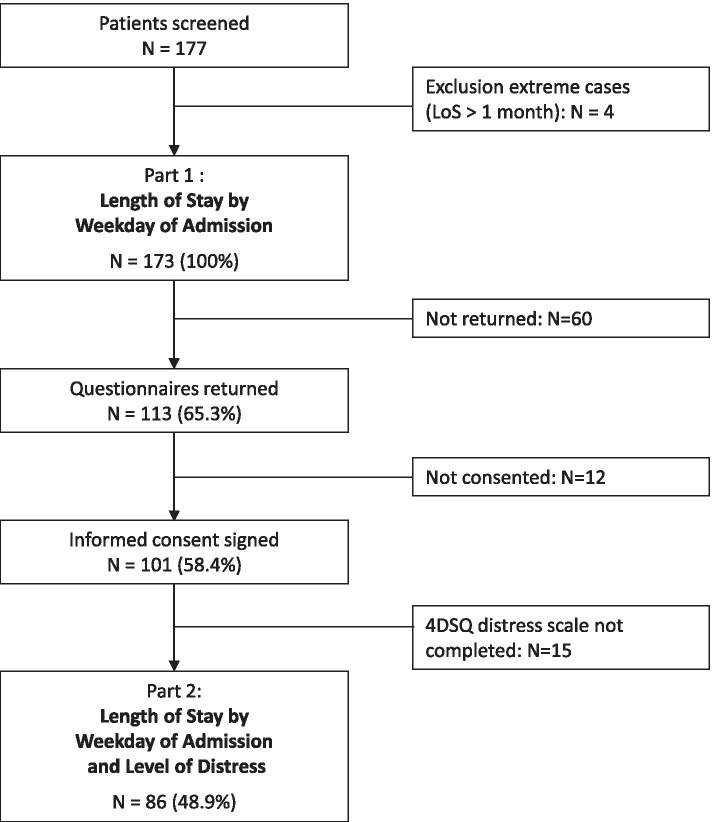
Study flow chart. N, number of cases; LoS, length of stay in hospital; 4DSQ, Four-Dimensional Symptom Questionnaire

The analyses in this study were based on two different samples (see Fig. 1). To analyse the effect of the weekday of admission on LoS (Part 1), we extracted data from the hospital’s electronic medical records using patient identification numbers. To record diagnoses related to specific causes of LBP (‘red flag’ pathologies), we investigated the medical records as well. As specific causes of LBP, we coded cancer, infection, trauma or inflammatory diseases such as spondylarthritis [16]. To analyse the influence of the weekday of admission and patient-reported distress on LoS (Part 2), we only used data from patients who signed the consent document. We did not include patients hospitalized for more than 1 month. In these cases, we assumed that significant complications occurred for reasons that cannot be explained by the initial LBP problem. The further use of routine health-related person data for this study was approved by the regional ethics committee (KEK ZH: 2020–01,465). All analyses were performed in accordance with guidelines and regulations from the regional ethics committee.

### Low back pain management in general medicine units

Patients with LBP admitted to medical units at Winterthur Cantonal Hospital receive care according to the clinical LBP pathway, with the aim of facilitating a patient-centred interdisciplinary approach (medicine, physiotherapy and nursing). The pathway requires that a specialized physiotherapist assess all patients. Physiotherapy was scheduled on the second day of hospitalization. Immediately after the first physiotherapy session, a consultation between the physiotherapist and the physician in charge should take place. This formal meeting focuses on the diagnosis, further inpatient procedures, and discharge management.

The general aim of the interdisciplinary approach is to achieve a mutual understanding of the pain problem and to set common goals together with the patient. This interdisciplinary procedure cannot be provided over the weekend. On weekends, interdisciplinary meetings are often not performed in general medicine units, as there is a reduction in the ratio of senior staff to patients on weekends [17]. For admissions on Friday afternoon, Saturday and Sunday, the physiotherapy assessment and the interdisciplinary meeting are postponed to Monday. On weekends, specialized physiotherapy assessments are not provided.

The physiotherapy assessment (60 min) is focused on hearing the patient’s full story regarding the cognitive and emotional experiences of their pain problem. Physiotherapists aim to explore patients’ beliefs, emotions, and stress responses associated with their current pain problem as well as their strategies for coping with pain and distress. Furthermore, the assessment addresses fear-related movements or avoidance behaviour. This functional analysis aims to identify maladaptive movements or postures, including extensive protective muscle activation or dysfunctional pain behaviour. In a collaborative process with the patient, physiotherapists explore patients’ ability to relax trunk muscles and to normalize pain-provocative postural and movement behaviours. Physiotherapists and physicians screen for specific causes of LBP (‘red flag’ pathologies).

Physicians are responsible for inpatient management, clinical diagnosis, diagnostic imaging, pharmacological treatment and invasive measures (e.g., surgical procedures). Attending physicians and nurses see patients daily during medical rounds, focusing on diagnosis, therapeutic procedures, and discharge management. Physiotherapists do not routinely take part in these rounds. However, information from the physiotherapy assessment is incorporated into the communication between the patient and health care professionals.

### Health-related personal data

We used the numeric rating scale (NRS, scale: 0–10) to measure average pain intensity over the last week. By means of the German version of the Roland Morris Disability Questionnaire (scale: 0–24), we measured back-specific function [18]. To evaluate patient-reported psychological distress, we used the distress scale of the German Four-Dimensional Symptom Questionnaire (4DSQ). The 4DSQ has been shown to be a valid self-reporting questionnaire to measure distress, depression, anxiety and somatization in patients treated in primary care [19]. The German version of the 4DSQ has previously been validated in a sample of multimorbid elderly people [20]. The questionnaire addresses the presence of symptoms during the last two weeks. Psychological distress was conceptualized as a direct manifestation of the effort people must exert to maintain their psychosocial homeostasis and social functioning when confronted with taxing life stress [19, 21]. In the four-dimensional model of the 4DSQ, distress is conceptualized as the most basic, most general or “normal” expression of psychological problems [19]. Higher scores on 4DSQ scales represent higher symptom severity.

### Statistical analysis

For the analysis in Part 1, we used a negative binomial model for LoS (dependent variable) with weekday of admission as a predictor. We tested the Poisson model against the negative binomial model using likelihood-ratio tests. Planned contrast was used to estimate the difference in log-expected LoS between the weekday of admission dichotomy (Friday/Saturday versus Sunday-Thursday). For Part 2, a negative binomial model was fitted for LoS (dependent variable) with weekday of admission, continuous patient-reported distress (4DSQ distress scale), and their interactions as covariates. We constructed the planned contrast of interest – the difference in (per unit) distress effect between group 1 (admissions Friday/Saturday) and the reference group (admissions Sunday-Thursday). This contrast represents the influence of the distress effect on LoS by day dichotomy (Friday/Saturday versus Sunday-Thursday). The level of statistical significance was set at 0.05. We performed all analyses using R statistical software [22].

## Results

### Part 1: length of stay in patients with LBP admitted to general medical units

During the 1-year period, 177 patients were screened. Four patients with LBP were hospitalized for more than 1 month (extreme cases: range = 31 to 54 days) and were excluded from the study. The mean age of the total study sample (Part 1: *N* = 173) was 66.10 years (standard deviation, SD = 16.21). Out of 173 patients, 103 were female (59.5%). In 20.8% of patients (*N* = 36), specific causes for LBP were diagnosed, most frequently fractures (*N* = 15), followed by cancer (*N* = 9), infection (*N* = 6), spondylarthritis (*N* = 4), and cauda equina (*N* = 2).

The overall mean LoS of all patients with LBP (*N* = 173) hospitalized in general medical units was 7.83 days (SD = 5.59). Table 1 provides descriptive statistics on LoS by weekday of admission in patients with LBP.

**Table 1:** Mean length of stay by weekday of admission in patients with low back pain hospitalized in medicine units (*N* = 173)

**Weekday of admission**	**N**	**Mean**	**SD**	**Min**	**Max**
Monday	32	7.28	4.59	3.00	24.00
Tuesday	28	5.89	3.98	2.00	17.00
Wednesday	21	7.05	3.71	1.00	15.00
Thursday	26	7.23	4.16	2.00	20.00
Friday	27	10.33	7.64	3.00	27.00
Saturday	19	10.58	7.24	4.00	27.00
Sunday	20	7.05	5.92	1.00	25.00

*N* Number of cases, *SD* Standard deviation, *min* Minimum, *max* Maximum

There was a difference in patients with LBP concerning the mean LoS between admissions on Friday and Saturday compared with other weekdays. The difference in the mean LoS between Saturday and Sunday was 3.5 days.

The distribution of the LoS variable in patients with LBP was right-skewed (skewness = 2.14), indicating asymmetry of sample distribution. The likelihood ratio test for nested models indicated that the negative binomial model, accounting for overdispersion, was more appropriate than the Poisson model. The likelihood ratio test for the global weekday effect was significant (likelihood ratio statistic = 17.56, df = 6, *p* = 0.007).

**Fig. 2:**
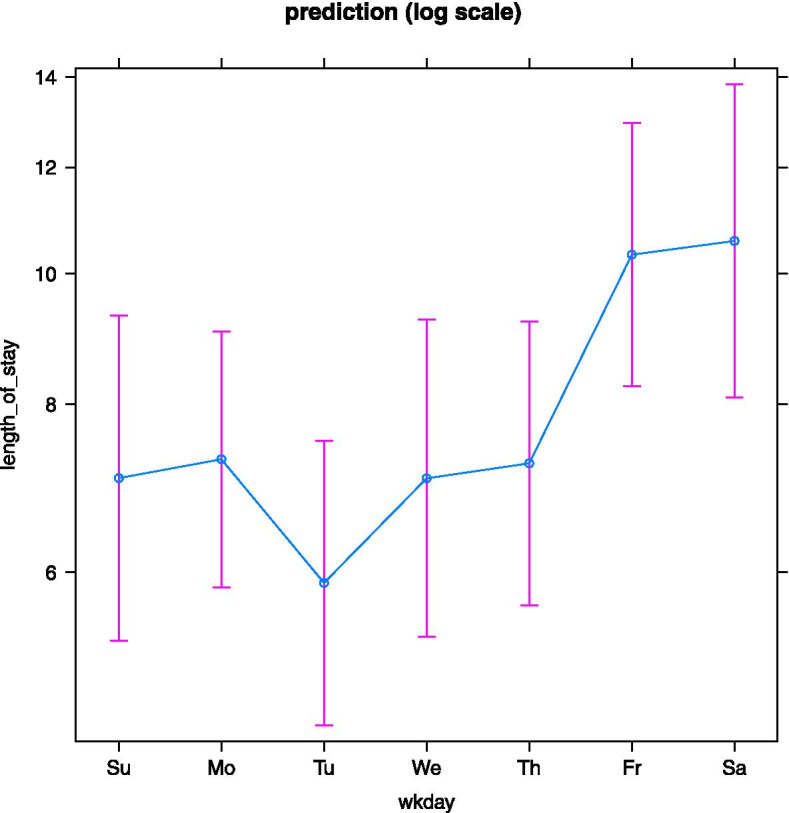
Predictions of length of stay by weekday of admission in patients with LBP. Error bars indicate 95% confidence intervals of the predicted LoS. Wkday, weekday of admission. LBP, low back pain

Figure 2 illustrates the LoS predictions on the log scale by weekday of admission in patients with LBP. Planned contrasts in the coefficients for weekdays showed that being admitted to the hospital with LBP on Friday or Saturday was associated with a significant difference in LoS compared with being admitted between Sunday and Thursday, where log ratio = 0.42 (95% CI = 0.21 to 0.63), corresponding to a rate ratio of 1.52 (95% CI = 1.24 to 1.87) on the response scale.

### Part 2: influence of weekday of admission and level of distress on length of hospitalization

Of the total study sample, 113 (65%) patients returned the questionnaire for collecting health-related personal data, and 101 patients (57%) signed the consent document. A complete set of data on patient-reported psychological distress (4DSQ distress scale) was available from 86 patients. This sample represents 48.9% of the total study sample (*N* = 173).

**Fig. 3:**
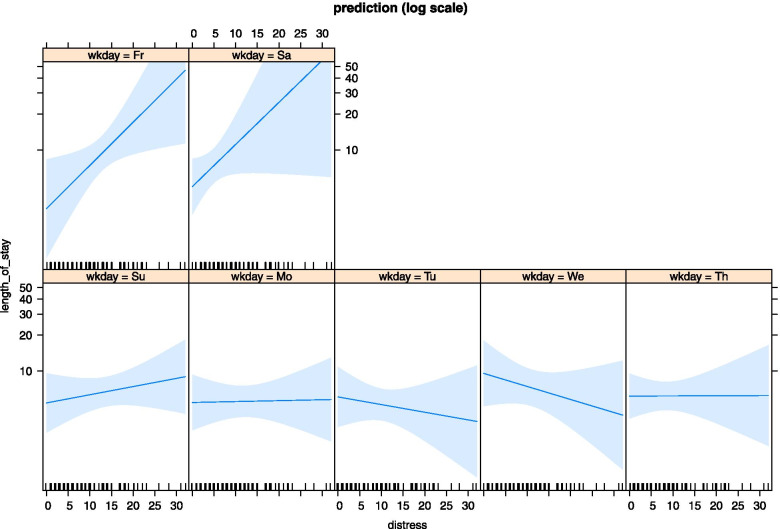
Distress predictor effect plots. Illustration of the moderation effect of weekday of admission on length of stay by patient-reported distress. The planned contrast between weekday dichotomies (Friday/Saturday versus Sunday-Thursday) is based on weekday-specific distress trends. The contrast is a linear function of the seven estimated slopes (contrast: -0.2 -0.2 -0.2 -0.2 -0.2 0.5 0.5). Wkday, weekday of admission

Figure 3 illustrates the estimated interaction effect (weekday of admission*distress) on LoS based on the negative binomial model in Part 2. The graph revealed that patient-reported distress had no or minimal influence on LoS when patients were admitted on Sunday, Monday, Tuesday, Wednesday, or Thursday. However, patients with LBP with high levels of self-reported distress admitted on Friday or Saturday stayed longer in the hospital than those reporting lower levels of distress. Planned contrast revealed that the difference in (per-unit) distress effect on LoS was significantly influenced by dichotomic day of admission (Friday/Saturday vs. Sunday-Thursday; contrast = 0.816 (95% CI = 0.03 to 1.60) on the response scale.

## Discussion

To the best of our knowledge, this study is the first to investigate the effect of weekday of admission and patient-reported levels of distress on LoS in patients with LBP in a primary care hospital. The results show that patients with LBP hospitalized on Friday or Saturday stayed more than 3 days longer in the hospital than those admitted on other weekdays. Although further research is necessary to explore these findings in more detail, it might be hypothesized that LoS could be prolonged on Fridays and Saturdays, as patients have to wait over the weekend for interdisciplinary LBP management to start. Furthermore, based on the observation that the effect of dichotomic weekdays of admission (Friday/Saturday vs. Sunday-Thursday) on LoS was moderated by patient level of distress, it might be hypothesized that the effect of delays in interdisciplinary LBP management on LoS is stronger in patients reporting high levels of distress.

We found significant differences in LoS in terms of the weekday of admission in patients with LBP. The potential ‘weekend effect’ on LoS has been shown in primary care hospitals. A meta-analysis based on 68 studies covering 640 million admissions disclosed that patients admitted during weekends stayed approximately 1 day longer than those admitted on other weekdays [14]. Delayed clinical management due to the weekend may have an impact on LoS in patients with LBP. However, we found a large difference in the mean LoS between Saturday and Sunday admissions (10.58 vs. 7.05 days) and not the known effect of delayed management on weekends, and LoS was also prolonged in patients admitted on Friday. This may indicate that patients’ waiting time for individualized care has a stronger impact on clinical management in patients with LBP.

To date, the effects of distress and weekday of admission on the LoS of inpatients with LBP have not been explored. An effect of distress on LoS has been found previously in patients undergoing elective joint replacement or joint arthroplasty. In this cohort, higher distress was associated with a longer LoS [23, 24]. Findings from these previous studies highlight the importance of addressing psychological factors before surgery and during hospitalization. Preceding studies on elective surgical procedures did not consider the influence of the weekday of admission on LoS. The reason might be that elective procedures are mainly conducted between Monday and Friday, and hospitalizations can be well planned. In patients with LBP and high levels of distress, the prolonged wait time through the weekend could have a negative impact on the individual’s suffering from pain and thus complicate inpatient management, including the planning of discharge management.

Our results further show that the effect of contrast in weekday of admission (Friday/Saturday vs. Sunday-Thursday) on LoS was moderated by patient-reported distress. This interaction effect suggests that higher distress is associated with a longer LoS if patients are hospitalized on Friday or Saturday. However, patient distress did not influence the LoS in patients admitted between Sunday and Thursday. It might be hypothesized that interdisciplinary LBP management was sufficient in managing LBP-related psychological distress under the condition that interdisciplinary multidimensional care started on the second day of hospitalization. This leads further to the hypothesis of patient trust as an important aspect of therapy outcome [25]. For patients with high levels of distress who are admitted on Friday or Saturday, waiting for a longer time to obtain comprehensive and convincing care might lead to less trust in LBP management and worse treatment outcomes, manifesting as a longer LoS.

### Implications

Although real-world interventions are necessary before we can derive clinical recommendations, the current study provides preliminary evidence for the importance of immediate patient-centred LBP management. Providing multidimensional and interdisciplinary LBP management might be easier in specialized units. However, not all hospitals have specialized inpatient units for patients with musculoskeletal pain. Our findings indicate that even with an interdisciplinary team including medical doctors, physiotherapists, and nurses, LoS can likely be shortened if the interdisciplinary approach is provided within the first days of hospitalization. However, in general medicine units at primary care hospitals, diagnostic and therapeutic services are usually reduced on weekends. Although organizational changes are difficult to implement, strategies to strengthen interdisciplinary LBP management during the weekend should be explored to reduce LoS in patients with LBP. Shortened LoS might offer the potential to reduce direct and indirect healthcare costs associated with LBP.

### Limitations

Some limitations concerning our study design should be considered. First, we used a retrospective rather than a prospective design. To investigate the value of a multidimensional physiotherapy assessment in inpatient care for patients with LBP, a prospective, controlled design is required. Second, the study was conducted in only one specific clinical setting, limiting the external validity of our results. Third, patient data were not measured immediately at the time of patient entry but after the first physiotherapy session. The patient questionnaire used for measuring distress aims to capture patient symptoms over the last two weeks. This should describe the distress level at the time of entry into the hospital; nevertheless, the delayed distribution of patient questionnaires could have distorted measures of psychological factors. Furthermore, the study included patients with nonspecific LBP and with specific causes of LBP. Possible differences between these patient groups were not explored. The heterogeneity of the study sample should be considered. Last, it should be taken into account that the negative binomial model on LoS was based on the data of the 86 patients who completed the 4DSQ. Nevertheless, we consider the estimations derived from this model for LoS with interactions as covariates as robust estimates of effects in the total study sample. Despite these limitations, it can be concluded that the LoS of patients with LBP is significantly prolonged if it is necessary to wait more than 2 days for interdisciplinary and multidimensional LBP management to begin. The influence of prolonged waiting time with a weekend admission might be strongest in patients reporting high levels of psychological distress.

### Future research

Inpatient care for patients with LBP remains a scarcely researched area. Our results suggest that the level of patient-reported distress influences the inpatient management of patients with LBP. Future research should further explore how patients’ psychological distress relates to therapeutic processes and treatment outcomes. Objective measures of individual suffering could help to systematically capture patients’ cognitive and emotional experiences of pain and to develop strategies for patient-centred LBP management in primary care hospitals. For this purpose, the Pictorial Representation of Illness and Self Measure (PRISM) could be useful when measuring patients’ individual experience of the relationship between their pain problems and their personhood [26–28]. The tool has been used in patients with rheumatoid arthritis to measure salient aspects of their personhood, which have been affected by the illness, and to estimate how this experience contributes to their suffering [29]. A systematic assessment of patients’ individual suffering would be helpful to evaluate LBP management and to develop patient-centred interventions. Furthermore, future research should investigate why patients with LBP are hospitalized in primary care hospitals. In our study, only approximately 20% had specific LBP with diagnosed serious spinal pathologies such as fractures, infections, cauda equina or cancer. For the other subgroup of patients with nonspecific LBP, it remains unclear why they were hospitalized and not treated in outpatient care settings. More knowledge about their characteristics and needs could help to improve inpatient care and to clarify possible care gaps in outpatient settings for this patient group. Finally, further research is needed to explore the influence of specific diagnoses and multimorbidity on LoS in patients with LBP. This could help identify possible subgroups of patients who need special attention at general medicine units, especially at weekends.

## Conclusions

It appears that patients with LBP are hospitalized significantly longer if they have to wait more than 2 days for interdisciplinary LBP management. It is likely that this particularly affects patients with LBP reporting high levels of psychological distress. Our study provides a platform to further explore whether interdisciplinary LBP management addressing patients’ multidimensional needs reduces LoS in primary care hospitals.

## Data Availability

To protect confidentiality, no raw data have been made available in any public repository. The datasets used and/or analysed during the current study are available from the corresponding author on reasonable request.

## Abbreviations

4DSQ: Four-Dimensional Symptom Questionnaire
LBP: Low back pain
LoS: Length of hospital stay
